# The Requirement of the C-Terminal Domain of GluA1 in Different Forms of Long-Term Potentiation in the Hippocampus Is Age-Dependent

**DOI:** 10.3389/fnsyn.2020.588785

**Published:** 2020-10-30

**Authors:** An Liu, Hong Ji, Qiaoyun Ren, Yanghong Meng, Haiwang Zhang, Graham Collingride, Wei Xie, Zhengping Jia

**Affiliations:** ^1^The Key Laboratory of Developmental Genes and Human Disease, Ministry of Education, School of Life Science and Technology, Jiangsu Co-Innovation Center of Neuroregeneration, Southeast University, Nanjing, China; ^2^Department of Physiology, Faculty of Medicine, University of Toronto, Toronto, ON, Canada; ^3^Neurosciences and Mental Health, The Hospital for Sick Children, Toronto, ON, Canada

**Keywords:** long-term potentiation, AMPA receptor, GluA1, C-terminal domain, high frequency stimulation, theta-burst stimulation, protein synthesis, ERK

## Abstract

Long-term potentiation (LTP) at glutamatergic synapses is an extensively studied form of long-lasting synaptic plasticity widely regarded as the cellular basis for learning and memory. At the CA1 synapse, there are multiple forms of LTP with distinct properties. Although AMPA glutamate receptors (AMPARs) are a key target of LTP expression, whether they are required in all forms of LTP remains unclear. To address this question, we have used our recently developed mouse line, GluA1^*C2KI*^, where the c-terminal domain (CTD) of the endogenous GluA1 is replaced by that of GluA2. Unlike traditional GluA1 global or conditional KO mice, GluA1^*C2KI*^ mice have no changes in basal AMPAR properties or synaptic transmission allowing a better assessment of GluA1 in synaptic plasticity. We previously showed that these mice are impaired in LTP induced by high-frequency stimulation (HFS-LTP), but whether other forms of LTP are also affected in these mice is unknown. In this study, we compared various forms of LTP at CA1 synapses between GluA1^*C2KI*^ and wild-type littermates by using several induction protocols. We show that HFS-LTP is impaired in both juvenile and adult GluA1^*C2KI*^ mice. The LTP induced by theta-burst stimulation (TBS-LTP) is also abolished in juvenile GluA1^*C2KI*^ mice. Interestingly, TBS-LTP can still be induced in adult GluA1^*C2KI*^ mice, but its mechanisms are altered becoming more sensitive to protein synthesis and the extracellular signal-regulated kinase (ERK) inhibitors compared to wild type (WT) control. The GluA1^*C2KI*^ mice are also differentially altered in several forms of LTP induced under whole-cell recording paradigms. These results indicate that the CTD of GluA1 is differentially involved in different forms of LTP at CA1 synapse highlighting the complexity and adaptative potential of LTP expression mechanisms in the hippocampus.

## Introduction

Long-term potentiation (LTP) at glutamatergic synapses is an extensively studied form of synaptic plasticity widely regarded as key mechanisms for learning and memory (Bliss and Collingridge, 1993; Malenka and Bear, 2004; Kandel et al., 2014). LTP has been most intensively investigated at the Schaffer collateral-commissural projection between CA3 and CA1 pyramidal neurons of the hippocampus. At these synapses, NMDA receptor-dependent LTP (NMDAR-LTP) is triggered by the activation of NMDARs and subsequent Ca^2+^ influx into the postsynaptic spine (Collingridge et al., 1983; Bliss and Collingridge, 1993). However, how the activation of NMDARs leads to long-lasting enhancement in synaptic efficiency remains unclear. Many studies have shown that the trafficking of AMPA receptors (AMPARs) represents a key mechanism in the expression of LTP (Davies et al., 1989; Malinow and Malenka, 2002; Bredt and Nicoll, 2003; Collingridge et al., 2004; Malenka and Bear, 2004; Shepherd and Huganir, 2007; Kessels and Malinow, 2009; Anggono and Huganir, 2012; Henley and Wilkinson, 2016; Diering and Huganir, 2018). Also, *in vitro* studies using recombinant receptors and peptides have shown that the C-TERMINAL DOMAIN (CTD) of GluA1, but not of GluA2, is required for activity-dependent synaptic delivery of AMPARs and expression of LTP (Hayashi et al., 2000; Shi et al., 1999, 2001; Boehm et al., 2006; Kessels and Malinow, 2009).

Genetic manipulations of endogenous AMPARs in mice have provided a powerful approach to address the specific role of individual endogenous receptor subunits in synaptic regulation and behavior. In earlier studies, it was found that LTP was impaired in GluA1 KO mice (Zamanillo et al., 1999), but could be induced in GluA2 and GluA3 KO mice (Jia et al., 1996; Meng et al., 2003; Toyoda et al., 2007), supporting the unique contribution of GluA1 in LTP expression. A recent study using floxed GluA mice combined with the use of recombinant receptors demonstrated that LTP can be established in the absence of all major AMPAR subunits, suggesting that AMPARs may not be a primary site for LTP expression (Granger et al., 2013; Huganir and Nicoll, 2013; Henley and Wilkinson, 2016). However, it is difficult to conclusively interpret the data from these KO studies because they were altered in baseline AMPAR complex (e.g., formation aberrant homomeric receptors), channel properties, and synaptic transmission (Jia et al., 1996; Andrásfalvy et al., 2003; Meng et al., 2003; Sans et al., 2003; Biou et al., 2008; Asrar et al., 2009; Lu et al., 2009; Zhou et al., 2011; Granger et al., 2013; Cao et al., 2018). Therefore, the extent to which endogenous AMPARs are involved in LTP expression remains unclear, especially under normal physiological conditions where these receptors are present.

We have recently generated a knock-in (KI) mouse line, called GluA1^*C2KI*^, where the CTD of endogenous GluA1 is replaced by that of GluA2 (Zhou et al., 2018). Unlike traditional GluA1 global or conditional KO mice, these mice have the expression of the GluA1 subunit, therefore avoiding the formation of the homomeric aberrant receptor complex. We have shown that GluA1^*C2KI*^ mice showed no impairments in AMPAR properties or long-term depression (LTD), but impairments in NMDAR-LTP induced by high-frequency stimulation (HFS, 100 Hz) in the hippocampal CA1 synapses. Because there are multiple forms of NMDAR-LTP at the CA1 synapse (Park et al., 2013), it is important to know whether the CTD of GluA1 equally contributes to these forms of LTP. Also, LTP mechanisms are subjected to developmental regulation (Yasuda et al., 2003; Palmer et al., 2004; Cao and Harris, 2012), therefore whether the involvement of GluA1 CTD also depends on the age of the animals is yet to be investigated. In this study, we compared hippocampal LTP induced by various protocols between GluA1^*C2KI*^ and their wild type (WT) littermates in both juvenile and young adult mice. We show that GluA1^*C2KI*^ mice are impaired in some, but not all forms of LTP, suggesting that the CTD of GluA1 differentially contributes to different forms of LTP.

## Materials and Methods

### Housing, Maintenance, and Use of the Mice

The GluA1^*C2KI*^ mouse model, where the CTD of GluA1 is replaced by the CTD of GluA2, was generated by using standard homologous recombination techniques in embryonic stem cells as described previously (Zhou et al., 2018). The GluA1^*C2KI*^ homozygous and WT littermates used for the present study were obtained from GluA1^*C2KI*^ heterozygous breeders. Both male and female mice (sex-balanced) were used in the present study. They were housed (2–5 mice per cage) on a 12 h/12 h light/dark cycle with food and water *ad libitum*. All experimental procedures were conducted during the light cycle following the guidelines of the Canadian Council on Animal Care (CCAC) and approved by the Animal Care Committee at the Hospital for Sick Children, Canada, and Southeast University, China. All experiments were performed blind to the genotype of the mice, that is, the mice were coded by an independent investigator before the experimentation and decoded after the completion of the experiments for data grouping and analyses.

### Slice Electrophysiology

All the electrophysiological recordings were done at the Schaffer collateral—commissural pathway in the hippocampus as previously described (Zhou et al., 2018). In brief, the mouse brains were removed and 360–400 μm brain slices prepared in ice-cold artificial cerebrospinal fluid (ACSF) saturated with 95% O_2_/5% CO_2_. ACSF contained (in mM): 120.0 NaCl, 3.0 KCl, 1.2 MgSO_4_, 1.0 NaH_2_PO_4_, 26.0 NaHCO_3_, 2.0 CaCl_2_, and 11.0 D-glucose. The slices were recovered at 28^o^C for at least 2 h before a single slice was transferred to a submersion chamber constantly perfused with 95% O_2_/5% CO_2_ saturated ACSF. The perfusion flow rate was maintained constant at 2 ml/min. In whole-cell recordings, ACSF also contained 100 μM picrotoxin and the recorded CA1 neurons were identified using an infrared differential interference contrast microscope (Zeiss Axioscope or Olympus X51). Synaptic response was elicited at 0.067 Hz for field potential recordings and 0.1 Hz for whole-cell currents, and recorded with glass pipettes (3–4 MΩ) filled with either ACSF (for field) or the intracellular solution (for whole-cell) containing (in mM) 130.0 CsMeSO_4_, 5.0 NaCl, 1 MgCl_2_, 0.05 EGTA, 10.0 HEPES, 3.0 Mg-ATP, 0.3 Na_3_GTP, and 5.0 QX-314 (pH 7.5; 280–300 mOsm). For field recordings, HFS-LTP was induced by four trains of HFS (100 Hz, 1 s) with an inter-train interval of 10 s, and TBS-LTP was induced by four trains of theta burst stimulations (five pulses at 100 Hz every 200 ms) with an inter-train interval of 10 s. For whole-cell experiments, cells were clamped at −65 mV throughout the recording except during LTP induction stimuli. Whole-cell LTP was induced either by two trains of HFS (100 Hz, 1 s, with an inter-train interval of 10 s, delivered under a current-clamp mode; referred to as HFS-CC-LTP) or by a pairing protocol (2 Hz, 90 s, delivered under a holding potential of 0 mV; referred to as paired-LTP). The age of mice was 13–15 postnatal days for juvenile mice and 5–6 postnatal weeks for young adult mice. LTP was calculated and statistically evaluated by comparing the mean values of the last 10 min of the recording and the mean values of the entire baseline. The drugs used included: Picrotoxin (Sigma–Aldrich, #R284556), D-AP5 (Tocris, #0106), AG-126 (APExBIO, #C4338), KN62 (APExBIO, #A8180), Anisomycin (APExBIO, #B6674) and Cycloheximide (APExBIO, #A8244). The use of these drugs is indicated in specific figure legends and was added to ACSF during the entire period of recording.

### Western Blot Analysis

Protein lysates were prepared from a hippocampal slice as previously described (Liu et al., 2016). Briefly, acute hippocampal slices were prepared, recovered, and treated with HFS or TBS in the same fashion as for electrophysiological recordings described above. Following the treatment, the slices were dissolved in ice-cold lysis buffer containing (in mM): 20 Tris-HCl (pH 7.5), 150 NaCl, 1 EDTA, 1 EGTA, 1% Triton X-100, 2.5 sodium pyrophosphate, 1 β-glycerophosphate, 1 Na_3_VO_4_, 20 NaF, and 1% protease inhibitor cocktail and phosphatase inhibitor (Roach) and kept at 4°C for 40 min and debris was removed by centrifugation at 14,000 *g* for 10 min. The protein samples were mixed with a 25% volume of 5× SDS loading buffer (250 mM Tris-HCl, 10% SDS, 0.5% bromophenol blue, 50% glycerol, 5% beta-mercaptoethanol, pH 7.4) for electrophoresis on an SDS–PAGE polyacrylamide gel and electrotransferred to a PVDF filter. The filter was then blocked with 5% dry milk in TBST (20 mM Tris-HCl, 9% NaCl, 1% Tween-20, pH 7.6) and incubated overnight at 4°C with suitable primary antibodies in TBST. Following washing and incubation with appropriate secondary antibodies, the filter was washed and developed using an enhanced chemiluminescence (Thermo Fisher Scientific, #34579) method of detection and analyzed using the AlphaEaseFC software according to manufacturer’s instruction. The amount of total protein loaded was controlled by normalizing each tested protein with anti-GAPDH immunoreactivity on the same blot. The antibodies used included: anti-p-ERK (Cell Signaling Technology, rabbit, #4370), anti-ERK (Cell Signaling Technology, rabbit, #4695), anti-GAPDH (Proteintech, mouse, 60008-1-lg), anti-GluA1-NTD (Cell Signaling Technology, rabbit, #8850), anti-GluA2-NTD (Millipore, rabbit, #MAB397), goat anti-rabbit (Genscript, #A00098), goat anti-mouse (Genscript, #A00160).

### Statistical Methods

All the averaged data in the graphs were stated as mean ± SEM and statistically evaluated by Student’s two-sided *t*-test for comparisons of two groups or one-way ANOVA for three groups followed by *post hoc* Holm-Sidak multiple comparison test. *p* < 0.05 was considered as significant (*p < 0.05, **p < 0.01, ***p < 0.001). Key mean ± SEM values, statistical parameters, and sample size are included in respective figure legends.

## Results

### The CTD of GluA1 Is Required for Both HFS- and TBS-LTP in Juvenile Mice

**Figure 1:**
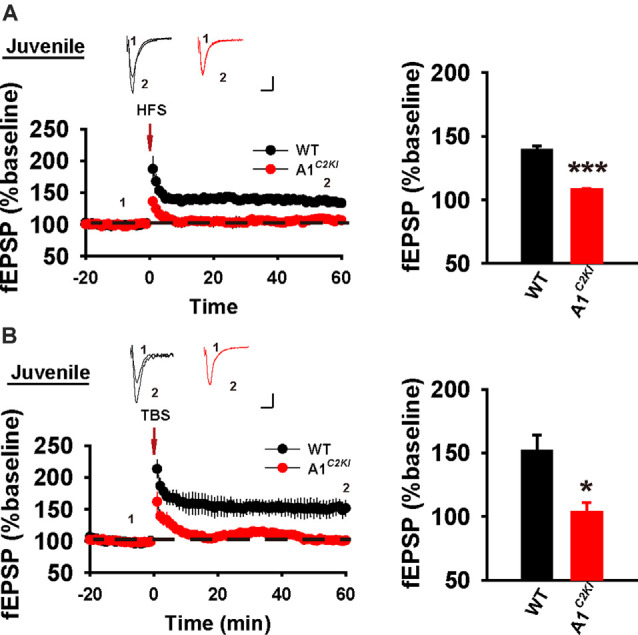
Absence of both high-frequency stimulation (HFS)- and theta-burst stimulation (TBS)–long-term potentiation (LTP) in juvenile GluA1^*C2KI*^ mice. **(A)** Absence of HFS-LTP in juvenile GluA1^*C2KI*^ mice (WT 139.30 ± 3.18%, *n* = 5 slices from three mice; GluA1^*C2KI*^ 107.86 ± 1.31%, *n* = 5 slices from three mice; *t*_(8)_ = 9.152, ***p < 0.001). **(B)** Absence of TBS-LTP in juvenile GluA1^*C2KI*^ mice (WT 151.52 ± 12.63%, *n* = 5 slices from three mice; GluA1^*C2KI*^ 103.54 ± 7.52%, *n* = 5 slices from three mice; *t*_(8)_ = 3.263, *p = 0.011). Scale bars: 0.15 mV/10 ms.

At the CA1 synapse, NMDAR-LTP can be induced either by HFS or theta-burst stimulation (TBS). Although the induction of both forms of LTP requires the activation of NMDARs, their expression mechanisms appear to be complex and partially distinct (Zhu et al., 2015). We previously showed LTP induced by either one train or multiple trains of 100 Hz HFS (referred to as HFS-LTP) is abolished in 3–4-week-old GluA1^*C2KI*^ mice (Zhou et al., 2018). However, whether LTP induced by TBS (referred to as TBS-LTP) is also affected in these mice is unknown. Also, although HFS-LTP is impaired in GluA1^*C2KI*^ mice, whether this effect is age-dependent remains to be determined. First, we examined LTP in juvenile mice (13–15 days old). As shown in Figure 1A, although HFS induced a persistent increase in field excitatory postsynaptic potential (fEPSP) in WT animals, this potentiation was not observed in GluA1^*C2KI*^ mice. Similarly, TBS induced LTP in WT mice, but not in GluA1^*C2KI*^ mice (Figure 1B). These results indicate that in juvenile mice the CTD of GluA1 is indispensable for both HFS- and TBS-LTP.

### The CTD of GluA1 Is Required for HFS-LTP, but Not TBS-LTP, in Adult Mice

**Figure 2:**
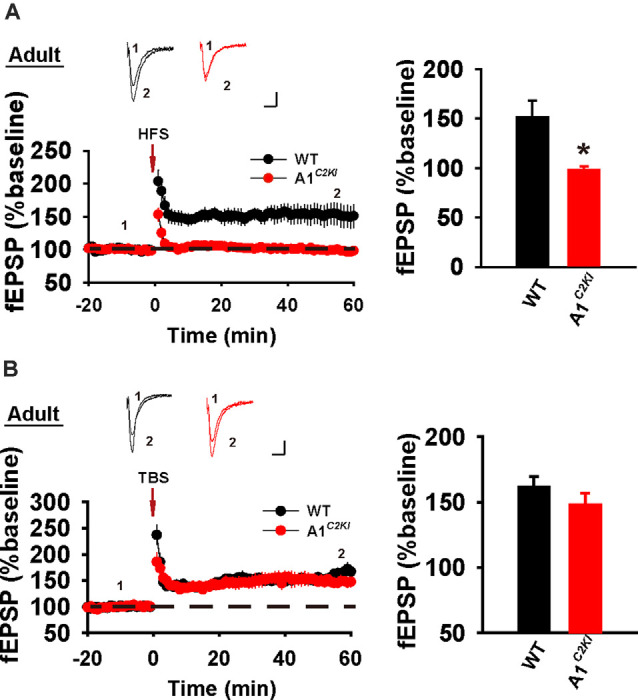
Abolished HFS-, but not TBS-LTP in adult GluA1^*C2KI*^ mice. **(A)** Absence of HFS-LTP in adult GluA1^*C2KI*^ mice (WT 151.74 ± 16.52%, *n* = 6 slices from three mice; GluA1^*C2KI*^ 92.21 ± 2.47%, *n* = 5 slices from three mice; *t*_(9)_ = 2.885, *p = 0.018). **(B)** Presence of TBS-LTP in adult GluA1^*C2KI*^ mice (wild type, WT 161.45 ± 8.06%, *n* = 5 slices from three mice; GluA1^*C2KI*^ 148.18 ± 8.95%, *n* = 5 slices from three mice; *t*_(8)_ = 1.102, *p* = 0.302). Scale bars: 0.15 mV/10 ms.

Next, we examined LTP in adult mice (5–6 weeks old). As shown in Figure 2A, HFS induced a persistent increase in fEPSP in WT animals, but this potentiation was not observed in GluA1^*C2KI*^ mice, indicating that the CTD of GluA1 is also indispensable for HFS-LTP in adult mice. However, TBS induced LTP with a similar magnitude in both WT and GluA1^*C2KI*^ mice (Figure 2B), suggesting that the CTD of GluA1 is not essential for TBS-LTP in adult mice. Therefore, although the requirement for the CTD of GluA1 for HFS-LTP persists in both juvenile and adult mice, its role in TBS-LTP is age-dependent.

### Mechanisms of TBS-LTP Are Altered in Adult GluA1^*C2KI*^ Mice

**Figure 3:**
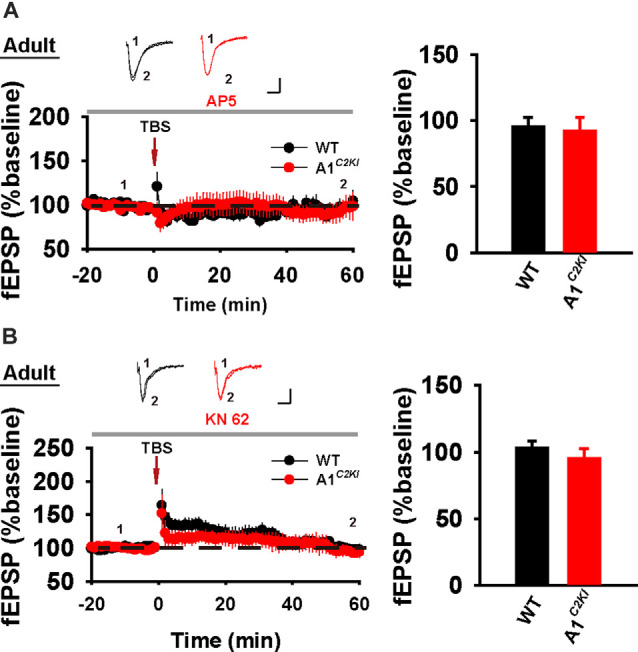
The requirement of NMDAR and CaMKII for TBS-LTP in adult GluA1^*C2KI*^ mice. **(A)** TBS-LTP in the presence of NMDAR inhibitor D-AP5 (50 μM) in adult WT and GluA1^*C2KI*^ mice (WT 95.47 ± 6.86%, *n* = 5 slices from three mice; GluA1^*C2KI*^ 91.84 ± 10.63%, *n* = 5 slices from three mice; *t*_(8)_ = 0.287, *p* = 0.782). **(B)** TBS-LTP in the presence of CaMKII inhibitor KN-62 (6 μM) in adult WT and GluA1C2KI mice (WT 103.11 ± 4.99%, *n* = 7 slices from four mice; GluA1^*C2KI*^ 95.20 ± 7.39%, *n* = 5 slices from three mice; *t*_(10)_ = 0.925, *p* = 0.377). Scale bars: 0.15 mV/10 ms.

**Figure 4:**
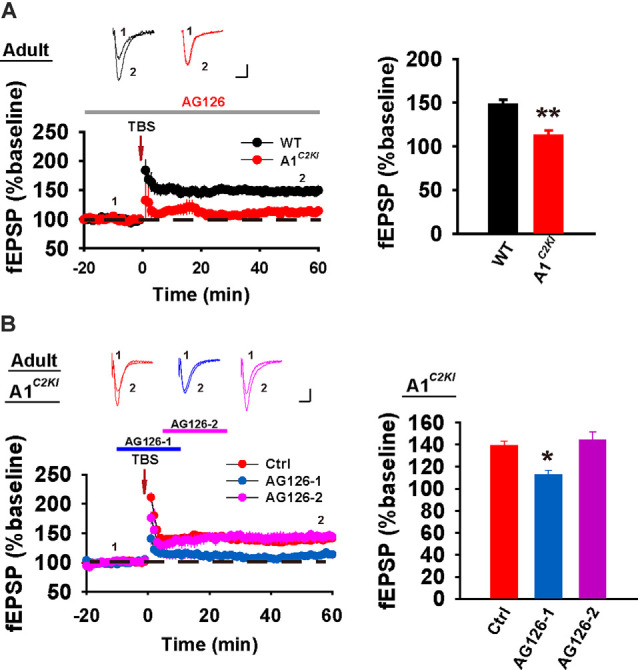
The requirement for ERK1/2 for TBS-LTP in adult GluA1^*C2KI*^ mice. **(A)** TBS-LTP in the presence of EKR1/2 inhibitor AG126 (50 μM) in adult WT and GluA1^*C2KI*^ mice (WT 148.00 ± 5.23%, *n* = 5 slices from three mice; GluA1^*C2KI*^ 112.29 ± 6.28%, *n* = 5 slices from three mice; *t*_(8)_ = 4.369, **p = 0.002). **(B)** Perfusion of AG126 during, but not after, the delivery of TBS impaired TBS-LTP in adult GluA1C2KI mice (*F*_(2,12)_ = 9.449, **p = 0.003; Ctrl 139.04 ± 4.21%, *n* = 5 slices from three mice; AG126-1 112.79 ± 4.10%, *n* = 5 slices from three mice, *p = 0.011 compared to Ctrl with *post hoc* Holm-Sidak multiple comparison tests; AG126-2 144.05 ± 7.41%, *n* = 5 slices from three mice, *p* = 0.529 compared to Ctrl with *post hoc* Holm-Sidak multiple comparison tests). Scale bars: 0.15 mV/10 ms.

**Figure 5:**
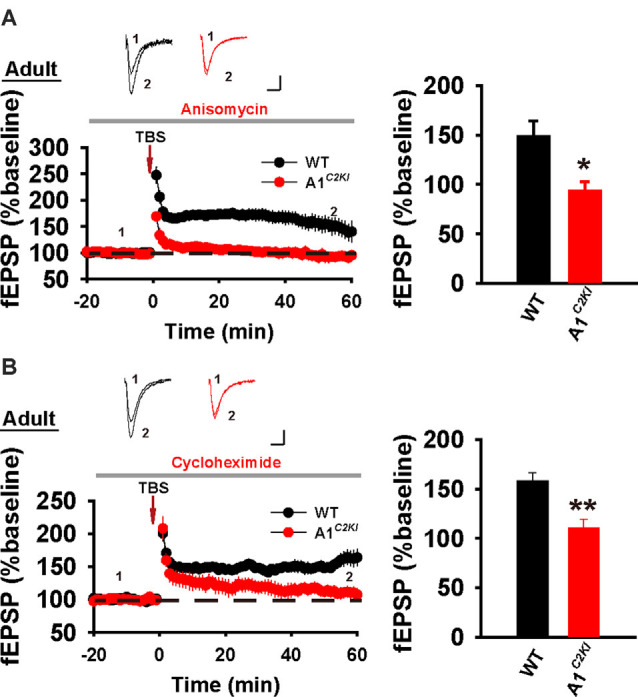
The requirement for protein synthesis for TBS-LTP in adult GluA1^*C2KI*^ mice. **(A)** TBS-LTP in the presence of protein synthesis inhibitor anisomycin (10 μM) in adult WT and GluA1^*C2KI*^ mice (WT 148.47 ± 15.55%, *n* = 6 slices from three mice; GluA1^*C2KI*^ 93.26 ± 9.44%, *n* = 5 slices from three mice; *t*_(9)_ = 2.877, *p = 0.018). **(B)** TBS-LTP in the presence of protein synthesis inhibitor cycloheximide (5 μM) in adult WT and GluA1^*C2KI*^ mice (WT 157.94 ± 8.47%, *n* = 6 slices from three mice; GluA1^*C2KI*^ 110.75 ± 8.30%, *n* = 5 slices from three mice; *t*_(9)_ = 3.935, **p = 0.003). Scale bars: 0.15 mV/10 ms.

Although TBS-LTP can be induced in adult GluA1^*C2KI*^ mice, its mechanisms may be different from those of WT animals. To test this possibility, we first examined the effect of the NMDAR antagonist AP5. As shown in Figure 3A, TBS-LTP was sensitive to AP5 in both WT and GluA1^*C2KI*^ mice, indicating that the LTP is NMDAR-dependent in both genotypes. Therefore, the induction mechanism remains intact in the mutant mice. We then tested the effect of KN62, a general inhibitor for the calcium/calmodulin-dependent protein kinase II (CaMKII), and showed that the inhibitor inhibited TBS-LTP in both WT and GluA1^*C2KI*^ mice, indicating that TBS-LTP requires activation of CaMKII in both genotypes (Figure 3B). We also tested the effect of tyrphostin AG-126, an inhibitor for extracellular signal-regulated kinase 1/2 (ERK1/2), a key signaling pathway implicated in several forms of LTP, particularly in the protein synthesis-dependent late-phase LTP (English and Sweatt, 1996; Winder et al., 1999; Kelleher et al., 2004a,b; Sweatt, 2004; Shalin et al., 2006; Costa-Mattioli et al., 2009; Kandel et al., 2014; Zhu et al., 2015; Vithayathil et al., 2017). As shown in Figure 4A, TBS-LTP was induced in WT mice in the presence of AG-126, suggesting that TBS-LTP induced in the present study is largely protein synthesis-independent (also referred to early-phase LTP). However, in GluA1^*C2KI*^ mice, TBS-LTP was significantly lower in the presence of AG-126 (Figure 4A). To test whether the effect of AG-126 was on the induction or maintenance of LTP, we perfused the drug either during the induction (10 min before and 10 after TBS) or after the induction of LTP (after TBS). As shown in Figure 4B, TBS-LTP was reduced by AG-126 application during, but not after, the delivery of TBS. To directly test the involvement of protein synthesis, we used two inhibitors for protein synthesis, anisomycin, and cycloheximide. In WT mice, neither anisomycin (Figure 5A) nor cycloheximide (Figure 5B) affected the magnitude of LTP (compare Figure 5 to Figure 2B without any inhibitors), confirming that TBS-LTP in WT mice does not require protein synthesis. In contrast, both anisomycin (Figure 5A) and cycloheximide (Figure 5B) significantly diminished TBS-LTP in GluA1^*C2KI*^ mice. These results suggest that TBS-LTP mechanisms in GluA1^*C2KI*^ mice have been modified to rely on ERK1/2 signaling and protein synthesis. Therefore, although TBS-LTP can be induced without the CTD of GluA1, its expression mechanisms are different from those of WT mice. Thus, the CTD of GluA1 appears to be particularly important for protein synthesis-independent, early-phase LTP.

### The CTD of GluA1 is Required for Whole-Cell LTP in Juvenile Mice

**Figure 6:**
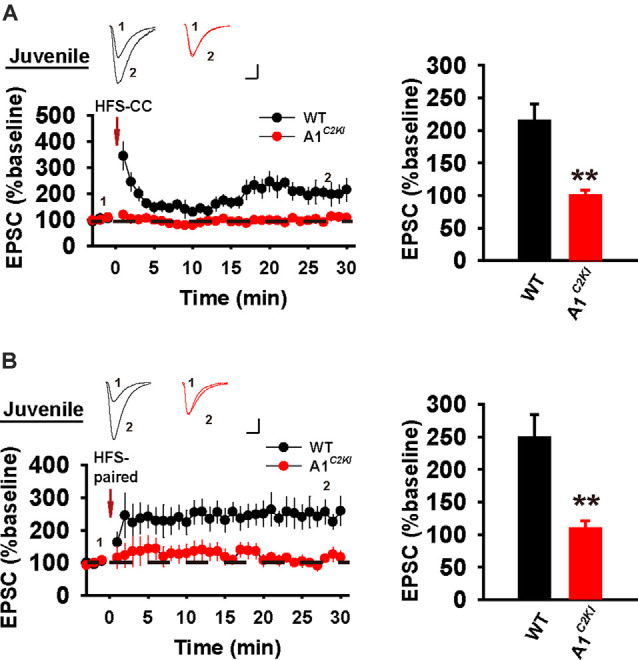
Absence of whole-cell HFS-CC-LTP and paired-LTP in juvenile GluA1^*C2KI*^ mice. **(A)** HFS-CC-LTP was induced in juvenile WT, but not in GluA1^*C2KI*^ mice (WT 214.41 ± 25.87%, *n* = 5 slices from three mice; GluA1^*C2KI*^ 99.98 ± 8.54%, *n* = 5 slices from three mice; *t*_(8)_ = 4.200, **p = 0.003). **(B)** Paired-LTP was induced LTP in WT, but not in juvenile GluA1^*C2KI*^ mice (WT 248.78 ± 35.70%, *n* = 5 slices from three mice; GluA1^*C2KI*^ 109.10 ± 11.95%, *n* = 5 slices from three mice; *t*_(8)_ = 3.710, **p = 0.006). Scale bars: 20 pA/10 ms.

In whole-cell recordings, LTP can be induced by the delivery of HFS under a current-clamp mode (referred to as HFS-CC-LTP, Figure 6A), which we used in our previous study (Zhou et al., 2018), or the delivery of moderate frequency stimulation paired with postsynaptic depolarization (referred to as paired-LTP, Figure 6B). In WT juvenile mice, both induction protocols induced a significant increase of the amplitude of excitatory postsynaptic currents (EPSCs) that persisted for at least 30 min and both forms of LTP were abolished in GluA1^*C2KI*^ mice (Figure 6). Therefore, similar to field potential recordings (Figure 1), whole-cell HFS-CC-LTP and paired-LTP in juvenile mice both require the CTD of GluA1.

### The CTD of GluA1 Is Required for Whole-Cell HFS-CC-LTP, but Not Paired-LTP in Adult Mice

**Figure 7:**
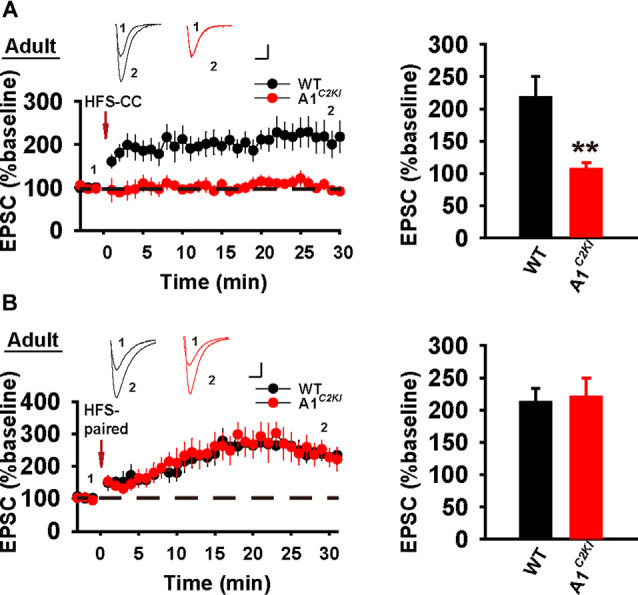
Absence of whole-cell HFS-CC-LTP, but the presence of paired-LTP in adult GluA1^*C2KI*^ mice. **(A)** HFS-CC-LTP was induced in adult WT, but not in GluA1^*C2KI*^ mice (WT 217.54 ± 32.50%, *n* = 6 slices from three mice; GluA1^*C2KI*^ 106.68 ± 10.24%, *n* = 6 slices from three mice; *t*_(8)_ = 3.254, **p = 0.009). **(B)** Paired-LTP was induced in both adult WT and GluA1^*C2KI*^ mice (WT 211.86 ± 21.31%, *n* = 5 slices from three mice; GluA1^*C2KI*^ 220.17 ± 29.25%, *n* = 5 slices from three mice; *t*_(8)_ = −0.230, *p* = 0.824). Scale bars: 20 pA/10 ms.

**Figure 8:**
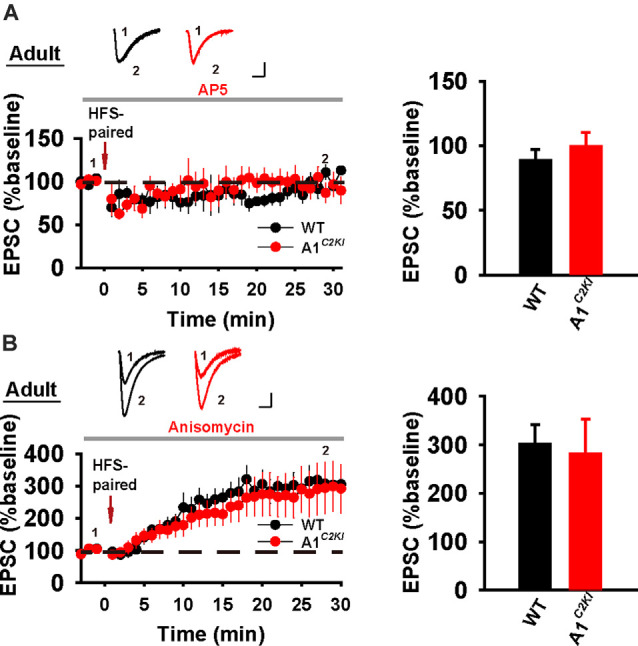
Whole-cell paired-LTP is depending on NMDARs, but not protein synthesis in adult GluA1^*C2KI*^ mice. **(A)** Paired-LTP was sensitive to D-AP5 (50 μM) in both adult WT and GluA1^*C2KI*^ mice (WT 88.86 ± 8.18%, *n* = 4 slices from three mice; GluA1^*C2KI*^ 99.41 ± 11.12%, *n* = 5 slices from three mice; *t*_(7)_ = −0.727, *p* = 0.491). **(B)** Paired-LTP in the presence of protein synthesis inhibitor anisomycin (10 μM) in adult WT and GluA1^*C2KI*^ mice (WT 302.15 ± 38.97%, *n* = 5 slices from three mice; GluA1^*C2KI*^ 281.81 ± 70.77%, *n* = 5 slices from three mice; *t*_(8)_ = 0.252, *p* = 0.808). Scale bars: 20 pA/10 ms.

We next examined whole-cell LTP in adult mice using these two induction protocols. As shown in Figure 7A, HFS-CC-LTP was induced in WT, but not in GluA1^*C2KI*^ mice, indicating that the CTD of GluA1 is also required for whole-cell HFS-CC-LTP in adult mice. However, paired- LTP was induced in both WT and GluA1^*C2KI*^ mice (Figure 7B). Also, this form of LTP in both WT and GluA1^*C2KI*^ mice was NMDAR-dependent (Figure 8A) and showed no differences in the presence of the protein synthesis inhibitor anisomycin (Figure 8B). These results suggest that the CTD of GluA1 is dispensable for whole-cell paired-LTP in adult mice.

### Enhanced ERK Activation and Increased GluA1 Protein Level Following TBS in Adult GluA1^*C2KI*^ Mice

**Figure 9:**
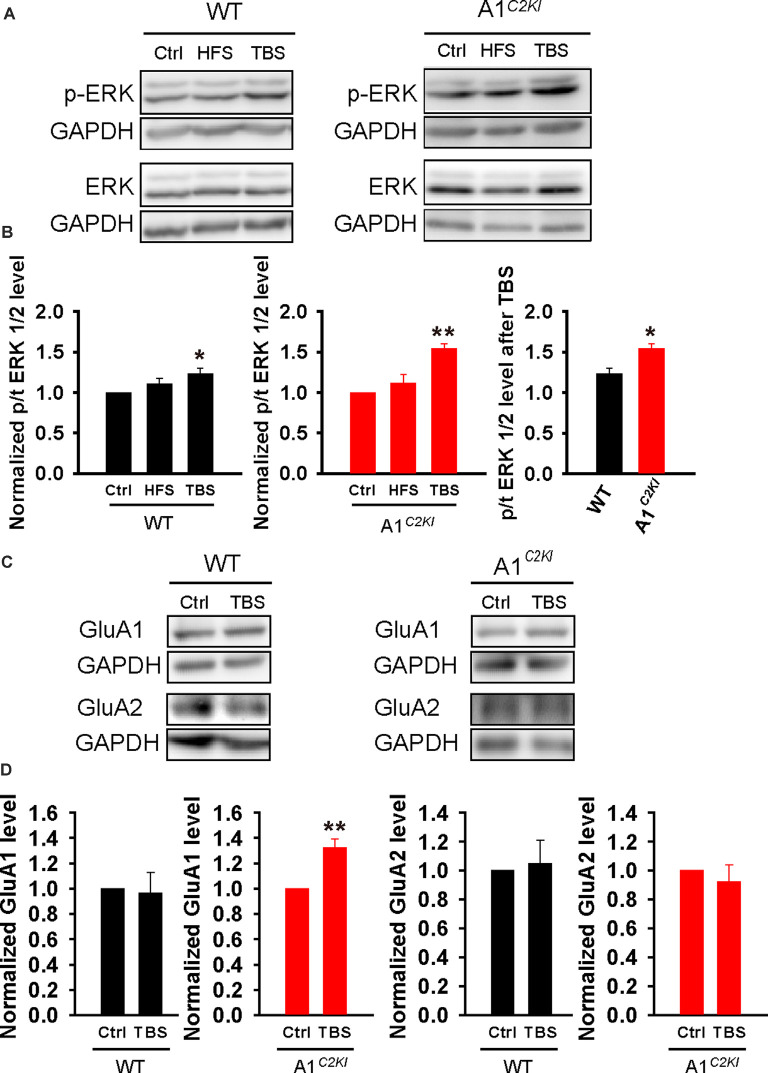
Enhanced extracellular signal-regulated kinase (ERK) activation and increased GluA1 protein level following TBS in adult GluA1^*C2KI*^ mice. **(A,B)** Sample western blots **(A)** and summary graphs **(B)** showing TBS, but not HFS, increased ERK1/2 phosphorylation in adult WT (*F*_(2,9)_ = 4.347, *p = 0.048; Ctrl 1.00 ± 0.00; HFS 1.11 ± 0.07, *p* = 0.204 compared to Ctrl with *post hoc* Holm-Sidak multiple comparison tests; TBS 1.23 ± 0.07, *p = 0.048 compared to Ctrl, and *p* = 0.276 compared to HFS with *post hoc* Holm-Sidak multiple comparison tests; *n* = 4 independent animals/experiments) and this increase was enhanced in adult GluA1^*C2KI*^ mice (*F*_(2,9)_ = 14.239, **p = 0.002; Ctrl 1.00 ± 0.00; HFS 1.11 ± 0.12, *p* = 0.334 compared to Ctrl with *post hoc* Holm-Sidak multiple comparison test; TBS 1.54 ± 0.06, **p = 0.002 compared to Ctrl with *post hoc* Holm-Sidak multiple comparison tests; *t*_(6)_ = 3.424, *p = 0.014 when comparing WT and GluA1^*C2KI*^ TBS; four independent animals/experiments). **(C,D)** Sample western blots **(C)** and summary graphs **(D)** showing increased GluA1 total protein level following TBS in adult GluA1^*C2KI*^, but not in WT mice (WT: Ctrl 1.00 ± 0.00; TBS 0.97 ± 0.16, *n* = four independent animals/experiments, *t*_(6)_ = 0.21, *p* = 0.841; GluA1^*C2KI*^: Ctrl 1.00 ± 0.00; TBS 1.32 ± 0.07, *n* = 5 independent animals/experiments, *t*_(8)_ = 4.486, **p = 0.002). The GluA2 protein level was not affected by TBS (WT: Ctrl 1.00 ± 0.00; TBS 1.05 ± 0.16, *n* = four independent animals/experiments, *t*_(6)_ = −0.298, *p* = 0.841; GluA1^*C2KI*^: Ctrl 1.00 ± 0.00; TBS 0.92 ± 0.12, *n* = 3 independent animals/experiments, *t*_(4)_ = 0.657, *p* = 0.547).

To explore the biochemical basis for the observation that TBS-LTP in GluA1^*C2KI*^ requires ERK activation and protein synthesis, we analyzed protein lysates from dissected CA1 regions with or without HSF/TBS stimulation. As shown in Figures 9A,B, TBS, but not HFS, induced a significant increase in phosphorylated (active) forms of ERK1/2 (p-ERK). This TBS-induced increase in p-ERK was significantly higher in GluA1^*C2KI*^ compared to WT mice (Figure 9B). The total ERK1/2 protein level was not altered by either HFS or TBS in WT or GluA1^*C2KI*^ mice. We also analyzed the protein level of GluA1 and GluA2 with or without TBS and found that GluA1 protein level was significantly increased in GluA1^*C2KI*^, but not in WT mice, following TBS treatment (Figures 9C,D). These results are consistent with the electrophysiological results that TBS-LTP in GluA1^*C2KI*^ is ERK- and protein synthesis-dependent (Figures 4, 5).

## Discussion

There is huge interest in understanding how AMPARs are involved in LTP because of its direct relevance to learning and memory (Bliss and Collingridge, 1993; Malenka and Bear, 2004; Kandel et al., 2014). Although it is generally agreed that trafficking of AMPARs into and out of the synapse is a key mechanism involved in LTP, the exact subunits and domains involved are not clear (Malinow and Malenka, 2002; Bredt and Nicoll, 2003; Collingridge et al., 2004; Malenka and Bear, 2004; Shepherd and Huganir, 2007; Kessels and Malinow, 2009; Anggono and Huganir, 2012; Huganir and Nicoll, 2013; Henley and Wilkinson, 2016). One limitation that is associated with the use of global or conditional GluA KO mice is that they are profoundly altered in AMPA receptor properties and basal synaptic transmission (Jia et al., 1996; Andrásfalvy et al., 2003; Meng et al., 2003; Lu et al., 2009; Granger et al., 2013; Cao et al., 2018), which greatly complicate the interpretation of the findings. In this study, we employed a recently generated mouse model where the CTD of the endogenous GluA1 is specifically replaced by that of GluA2. We previously demonstrated that these mice are impaired in hippocampal LTP induced by HFS (Zhou et al., 2018). In this study, we have extended LTP analysis with additional protocols in both juvenile and adult mice and shown that the involvement of the CTD of GluA1 in LTP is age- and induction protocol-dependent.

First, we showed that HFS-LTP is abolished in GluA1^*C2KI*^ mice consistent with previous results (Zhou et al., 2018). Also, this abolition of LTP applies to both juvenile and young adult mice. Furthermore, HFS-LTP is absent in both field and whole-cell recording conditions. These results indicate that the CTD of GluA1 is essential for HFS-LTP under various physiological conditions and developmental stages. These results are also consistent with the data obtained from GluA1 KO mice where LTP induced by tetanic stimulation (100 Hz, 1 s), an induction protocol similar to the HFS protocols used in the present study, is impaired (Zamanillo et al., 1999).

Second, we showed that TBS-LTP in GluA1^*C2KI*^ mice is age-dependent. In juvenile animals, TBS-LTP is abolished in GluA1^*C2KI*^ mice, whereas in adult mice, TBS-LTP is still present in GluA1^*C2KI*^ mice. These results suggest that the requirement for the CTD of GluA1 in TBS-LTP is developmentally regulated, being essential in early development, but maybe compensated by additional mechanisms in adult mice. These results are as per the results obtained from GluA1 KO mice where some forms of LTP can still be established in these mice (Hoffman et al., 2002; Jensen et al., 2003; Shimshek et al., 2017).

It is important to emphasize that although TBS-LTP can be induced in GluA1^*C2KI*^ adult mice, its mechanisms are altered. In both WT and GluA1^*C2KI*^ adult mice, TBS-LTP is sensitive to AP5 indicating that it is NMDAR-dependent. Also, TBS-LTP in both genotypes is affected by KN-62, and therefore requires activation of CaMKII. However, while the TBS-LTP in WT mice is insensitive to anisomycin or cycloheximide, TBS-LTP in GluA1^*C2KI*^ adult mice is inhibited by these drugs, and therefore it is dependent on new protein synthesis. The dependence of TBS-LTP on protein synthesis in GluA1^*C2KI*^ adult mice is also supported by the results that the inhibition of ERK1/2, a key protein kinase involved in protein synthesis and late-phase LTP (Kelleher et al., 2004b; Costa-Mattioli et al., 2009), impairs TBS-LTP in GluA1^*C2KI*^, but not in WT adult mice. Consistent with these recording data, TBS, but not HFS, induces activation of ERK1/2, and this TBS-induced ERK1/2 activation is significantly enhanced in GluA1^*C2KI*^, compared to WT adult mice. It is possible that TBS used in the present study induces both protein synthesis-dependent and -independent pathways, but under normal physiological conditions, the protein-independent pathways such as protein phosphorylation and trafficking of existing AMPARs, is the predominant mechanism underlying LTP expression and this mechanism requires the CTD of GluA1 and CaMKII, but not ERK1/2. However, in GluA1^*C2KI*^ mice, TBS induces a higher level of ERK1/2 activation compared to WT, and this activates ERK1/2 downstream signaling pathways, which in turn initiate new protein synthesis and overcome the defect in protein delivery by overproducing GluA1 and/or other LTP related proteins. The fact that the application of AG-126 during, but not after TBS, blocks TBS-LTP suggests that ERK1/2 is transiently activated during the induction period and that its sustained activation may not be necessary for LTP maintenance. How the ERK1/2 activation leads to new protein synthesis in GluA1^*C2KI*^ mice is unknown, but it is well established that ERK1/2 is an upstream regulator of protein synthesis signaling molecules and regulatory factors, including the mTOR pathway and the translational initiation factors, that are involved in protein synthesis-dependent synaptic plasticity (Kelleher et al., 2004b; Costa-Mattioli et al., 2009). It would be interesting to examine whether these signaling proteins are altered following TBS, which would allow new protein synthesis more easily in GluA1^*C2KI*^ mice.

Finally, it is important to note that the results from whole-cell recordings are largely consistent with those of field potential recordings. In juvenile GluA1^*C2KI*^ mice, whole-cell HFS-CC-LTP and paired-LTP are both impaired, underscoring the significance of the CTD of GluA1 in LTP expression at this developmental stage. In adult GluA1^*C2KI*^ mice, whole-cell HFS-CC-LTP is also impaired in GluA1^*C2KI*^ mice, supporting the essential role of GluA1 CTD in the expression of HFS-CC-LTP throughout the lifetime of the mice. However, in adult GluA1^*C2KI*^ mice, whole-cell paired-LTP is still present. A pairing protocol may elicit multiple signaling processes, as a TBS protocol in the field potential recordings, that overcomes the requirement for the GluA1 CTD and allows the expression of LTP. Although this form of LTP is NMDAR-dependent and protein synthesis-independent, as in WT mice, further studies are needed to investigate whether it is mechanistically distinct.

A recent study by Díaz-Alonso et al. (2020) also tested the involvement of the GluA CTD in hippocampal CA1 LTP by using a gene replacement in a KO background and generating KI mice lacking the CTD of GluA1 *via* a CRISPR approach and found that LTP can be induced using a pairing protocol in whole-cell recordings in these mice. This result seems consistent with our data that paired-LTP can be induced in GluA1^*C2KI*^ mice as shown in this study. Since Díaz-Alonso et al study did not report data on field LTP or whole-cell HFS-CC-LTP, which show most significant impairments in GluA1^*C2KI*^ mice and GluA1 global KO mice (Zamanillo et al., 1999; Zhou et al., 2018; including the current study), no direct comparison between our and Díaz-Alonso et al study can be made under these conditions. Besides, the methodologies used in our and Díaz-Alonso et al study were also different that could contribute to the discrepancy (e.g., in spatial learning and memory). First, the gene replacement strategy in Díaz-Alonso et al study involves overexpression of exogenous receptors in a KO background and therefore may not represent the behavior of the endogenous receptors. Second, instead of the CTD replacement in our GluA1^*C2KI*^ mice which produce a full-length receptor, the GluA1 mutant mice in Díaz-Alonso et al study deleted the entire CTD of GluA1 plus the insertion of a HA tag, which resulted in a truncated form of GluA1. This truncation/insertion could potentially alter the confirmation/structure of the receptor thus confounding the analysis of LTP and behavior.

In summary, by using our recently created mouse strain where the CTD of the endogenous GluA1 is replaced by that of GluA2, we show that the CTD of GluA1 is differentially required for different forms of LTP and this effect is regulated by developmental stages. Our results are consistent with previous genetic studies using GluA1 global KO and phosphorylation site KI mice showing that GluA1 is indispensable for some, but not all, forms of hippocampal LTP (Zamanillo et al., 1999; Hoffman et al., 2002; Jensen et al., 2003; Lee et al., 2003; Shimshek et al., 2017). These results highlight the complexity of LTP expression mechanisms at the AMPAR level. This is not surprising given the diverse posttranslational modifications existing at the CTD of AMPARs (Diering and Huganir, 2018). It would be interesting to elucidate how the CTD of GluA1 differentially regulates these forms of LTP and how they might be altered in various brain disorders.

## Data Availability Statement

The raw data supporting the conclusions of this article will be made available by the authors, without undue reservation.

## Ethics Statement

The animal study was reviewed and approved and all experimental procedures were conducted in accordance with the guidelines of the Canadian Council on Animal Care (CCAC) and approved by the Animal Care Committee at the Hospital for Sick Children, Canada and Southeast University, China.

## Author Contributions

ZJ, AL, and WX designed the experiments. AL, HJ, QR, YM, and HZ performed the experiments and analyzed the data. ZJ, AL, and GC wrote the article. All authors contributed to the article and approved the submitted version.

## Conflict of Interest

The authors declare that the research was conducted in the absence of any commercial or financial relationships that could be construed as a potential conflict of interest.

## Abbreviations

AMPARs: AMPA receptors
CaMKII: calcium/calmodulin-dependent protein kinase II
CTD: c-terminal domain
ERK: the extracellular signal-regulated kinase
fEPSP: field excitatory postsynaptic potential
EPSC: excitatory postsynaptic current
GluA1^*C2KI*^: GluA1 CTD replaced by GluA2 CTD
KO: knock out
LTP: long-term potentiation
HFS: high-frequency stimulation
NMDARs: NMDA receptors
TBS: theta-burst stimulation
WT: wild type.
